# Translational Gaps in Assessing the Obesogenic Effects of Herbicides: A Scoping Review

**DOI:** 10.3390/jox16050173

**Published:** 2026-09-13

**Authors:** Marco Antonio Ramírez-Vargas, Cristian Oswaldo Bravo-Nava, Ma. Elena Moreno-Godínez, Gerardo Huerta-Beristain, Eugenia Flores-Alfaro, Guillermina Vences-Velázquez, José Ángel Cahua-Pablo

**Affiliations:** Laboratorio De Toxicología y Salud Ambiental, Facultad de Ciencias Químico-Biológicas, Universidad Autónoma De Guerrero, Av. Lázaro Cárdenas s/n, Chilpancingo de los Bravo 39087, Guerrero, Mexico; 17394368@uagro.mx (C.O.B.-N.); emoreno20@hotmail.com (M.E.M.-G.); 14478@uagro.mx (G.H.-B.); eugeniaflores@uagro.mx (E.F.-A.); guillerminavences@uagro.mx (G.V.-V.); jcahua@uagro.mx (J.Á.C.-P.)

**Keywords:** translational toxicology, environmental obesogens, adipogenesis, endocrine-disrupting chemicals, reverse dosimetry

## Abstract

Obesity is a disease characterized by excessive accumulation of adipose tissue. It is a multifactorial condition arising from complex interactions among genetic background, lifestyle-related factors (such as physical inactivity, socioeconomic and cultural determinants), and environmental factors, including dietary patterns and exposure to environmental pollutants. In this context, exposure to environmental pollutants has been considered an independent risk factor for the development of obesity. However, the evidence linking herbicide exposure and obesity is limited. Therefore, the present scoping review aimed to map the available evidence regarding the obesogenic effects of herbicides across *in vitro*, *in vivo*, and epidemiological models, evaluate their translational concordance, and identify methodological gaps in the available literature. A systematic literature search was conducted in PubMed, Web of Science, and Scopus to identify studies reporting the effect of herbicide exposure and obesity-related endpoints. The search was limited to March 2026. A total of 24 studies fully met our eligibility criteria and were classified into *in vitro*, *in vivo*, and epidemiological evidence categories. The findings revealed a substantial disconnect across the different levels of evidence. In both *in vitro* and *in vivo* studies, the concentrations and doses tested often did not reflect realistic exposure scenarios, thereby limiting the applicability of these findings to human health risk assessment. In contrast, epidemiological studies frequently relied on obesity indicators with limited sensitivity and specificity, such as body mass index (BMI). Overall, the evidence suggests limited external validity in population-based studies and the arbitrary selection of concentrations and doses in experimental models.

## 1. Introduction

Obesity is a chronic, complex, and multifactorial disease characterized by excessive accumulation of adipose tissue. Increased adiposity, a key hallmark phenotype of this clinical condition, impairs function across multiple organ systems. Obesity is considered an independent risk factor for the development of cardiometabolic diseases, several types of cancer, and immune-related disorders [1]. In the last five decades, the prevalence of excess adiposity, including both overweight (body mass index [BMI] 25.0–29.9 kg/m^2^) and obesity (BMI ≥ 30 kg/m^2^), has gradually increased worldwide. Although obesity is most prevalent in high-income countries, its burden has increased across all world regions. It has been estimated that, between 1990 and 2020, the prevalence of adult obesity increased by 104.9% among women and 155% among men [2].

The development of obesity involves a complex interplay of genetic, sociocultural, economic, lifestyle (including dietary habits, physical activity, sleep patterns, and sedentarity), and psychosocial factors. Exposure to environmental obesogens has been suggested as a potential risk factor associated with the development of obesity. Accordingly, several epidemiological and experimental studies have shown that exposure to environmental contaminants is linked to the onset and progression of chronic diseases, including obesity [3]. Pesticides are considered a heterogeneous group of chemical substances used for pest and vector-borne disease control. Environmental and occupational pesticide exposures are recognized as a risk factor for a wide range of adverse health outcomes, including cardiometabolic, immunological, neurological, developmental, and reproductive disorders, as well as various types of cancer [4]. The assessment of pesticide-exposure-induced health effects is traditionally focused on insecticides. However, herbicide use is higher than insecticide use. According to estimations from the Food and Agriculture Organization (FAO), approximately 759,403 tons of insecticides and 2,009,099 tons of herbicides were used worldwide in 2023 [5]. For this reason, there is a need for a more comprehensive evaluation of the potential health effects associated with herbicide exposure, including its possible role in the development of obesity.

Toxicology research is considered translational because it assesses the health effects of a wide range of potentially hazardous agents. Despite significant efforts to integrate findings from *in vitro* studies, *in vivo* experiments, and epidemiological evidence to comprehensively characterize health risks, the available evidence remains heterogeneous. Differences and limited concordance among methodological approaches across these study designs complicate the integration and interpretation of findings within a unified risk assessment framework [6,7]. Scoping reviews offer an opportunity to evaluate methodological heterogeneity across experimental models and their concordance with epidemiological evidence. They also enable systematic mapping of evidence assessing the relationship between herbicide exposure and the development of obesity. This review seeks to characterize experimental designs, evaluate translational concordance of tested doses, identify methodological gaps, and propose a strategic framework to inform future toxicological and epidemiological research.

## 2. Material and Methods

### 2.1. Evidence Searches

A scoping review was performed following the guidance of the PRISMA extension for scoping reviews [8] (PRISMA-ScR checklist is presented in Supplementary Material S1) and the recommendations of the Joanna Briggs Institute (JBI) methodology for scoping reviews [9,10]. A protocol was developed and registered in the Open Science Framework (OSF; https://osf.io/muby4/files/d475e; created on 4 April 2026).

### 2.2. Search Strategy and Information Sources

A systematic literature search was conducted using PubMed, Web of Science, and Scopus. All relevant articles published up to 31 March 2026, were retrieved. The search algorithms captured variations of the following conceptual domains: “obesogens,” “herbicides,” “adipogenesis,” “adipocyte maturation/differentiation,” “adipose tissue hyperplasia/hypertrophy,” and “adipose mass”. In addition, the literature cited in each record recovered was analyzed to identify additional records. The search strategies and terms used for each database are provided in Supplementary Material S2.

### 2.3. Selection Criteria

This scoping review included a search for original studies evaluating the impact of herbicide exposure on obesity-related and obesogenic markers. Original studies conducted in experimental models (*in vitro* and *in vivo*) and epidemiological studies were considered eligible for inclusion.

Inclusion criteria

Original English-language studies evaluating the obesogenic potential of herbicides were collected. The following endpoints were considered relevant outcomes:

- Preadipocyte proliferation and differentiation.
- Activation of key adipogenic regulators, including but not limited to Sterol Regulatory Element-Binding Proteins (SREBPs), CCAAT/enhancer-binding proteins (C/EBPs), and Peroxisome Proliferator-Activated Receptor gamma (PPARγ).
- Increases in overall adiposity or fat mass.
- Clinical indicators used for the diagnosis of obesity, including but not limited to body mass index (BMI) and waist circumference.
- Histomorphometric parameters of adipose tissue used to distinguish adipocyte hyperplasia from adipocyte hypertrophy.

Exclusion Criteria

Studies meeting any of the following criteria were excluded:

- Publication type: narrative, systematic, or scoping reviews; meta-analyses; letters to the editor; editorials; commentaries; and conference abstracts that did not provide a complete primary dataset.
- Study design: exclusively in silico studies (e.g., computational modeling or molecular docking) without subsequent biological validation in either *in vitro* or *in vivo* models.
- Exposure focus: studies evaluating other endocrine-disrupting chemicals (e.g., plasticizers or heavy metals) or other classes of pesticides (e.g., insecticides or fungicides) within complex mixtures in which the independent effect of herbicide exposure could not be distinguished.
- Duplicate populations: when multiple publications from the same research group reported findings from the same study population or cohort, only the most recent publication or the study providing the most comprehensive dataset was retained.

### 2.4. Study Selection

All retrieved records were imported into Zotero v. 9.0.6 reference management software, where duplicate entries across databases were identified and removed. Subsequently, two independent reviewers (COBN and MARV) applied the study selection criteria through a peer-review screening process. Any discrepancies regarding study eligibility were resolved by consensus.

### 2.5. Data Charting

A standardized data-charting form was developed to extract key variables tailored to the specific type of evidence being evaluated. Beyond author and publication year, the following data were extracted:

- *In vitro* studies: Cell line, tested herbicide, purity grade (analytical vs. commercial formulation), tested concentrations, exposure duration, and targeted adipogenesis biomarkers.
- *In vivo* studies: Animal model, age and sample size, tested herbicide and purity grade, administered dose and exposure route, duration of exposure, and obesity-related outcomes.
- Epidemiological studies: Study design, geographic location (country), sample size, participant demographics, methods for assessing herbicide exposure (e.g., biomonitoring vs. questionnaires), and clinical obesity outcomes measured

### 2.6. Risk of Bias

The risk of bias of the included studies was assessed to identify methodological shortcomings that could affect the synthesis and interpretation of evidence within a translational toxicology framework. Risk-of-bias assessment for epidemiological studies was conducted using the OHAT Risk of Bias Tool [11]. Four critical domains were designated as key criteria for determining study reliability: (1) selection bias (appropriateness of comparison groups), (2) confounding bias (adjustment for important confounding/modifying variables), (3) detection bias in exposure characterization, and (4) detection bias in outcome assessment. In the OHAT Risk of Bias Tool, these key criteria for assessing the risk of bias are indicated in bold. The included studies were evaluated based on their ratings across key and secondary domains, which were categorized as definitely low risk (++), probably low risk (+), probably high risk (-), or definitely high risk (--). Ultimately, all included epidemiological studies were classified into three tiers as follows: Tier 1 (High Quality): Studies received a “definitely low risk of bias” (++) rating in all four key criteria and had no “probably high risk” (-) or “definitely high risk” (--) ratings in any of the secondary domains. Tier 2 (Moderate Quality): Studies did not meet the stringent criteria for Tier 1 but lacked critical methodological flaws. A study was classified as Tier 2 if it received a “probably high risk” (-) rating in either a key criterion or a secondary domain, provided no critical flaws were present. Tier 3 (Low Quality): Studies were automatically assigned to this tier if they demonstrated a critical methodological flaw, defined as receiving a “definitely high risk of bias” (--) rating in at least one of the key criteria. In contrast, animal studies were evaluated using SYRCLE’s Risk of Bias Tool [12], a standardized instrument designed specifically for *in vivo* research. Three independent reviewers assessed the risk of bias for all included *in vivo* and epidemiological studies, resolving any discrepancies through consensus.

### 2.7. Data Synthesis and Presentation

The charted data were summarized narratively and presented using tabular arrays, categorized by the level of evidence (*in vitro*, *in vivo*, and epidemiological). The charted data were summarized narratively and presented using tabular arrays, categorized by the level of evidence (*in vitro*, *in vivo*, and epidemiological).

### 2.8. Translational Concordance:

Information derived from *in vivo* and *in vitro* models was considered concordant with human evidence when the experimental doses were environmentally relevant. Specifically, experimental exposures were classified as realistic when they were directly associated with established toxicological risk parameters, including, but not limited to, regulatory values such as Acceptable Daily Intake (ADI), Tolerable Daily Intake (TDI), or Reference Dose (RfD).

## 3. Results

**Figure 1:**
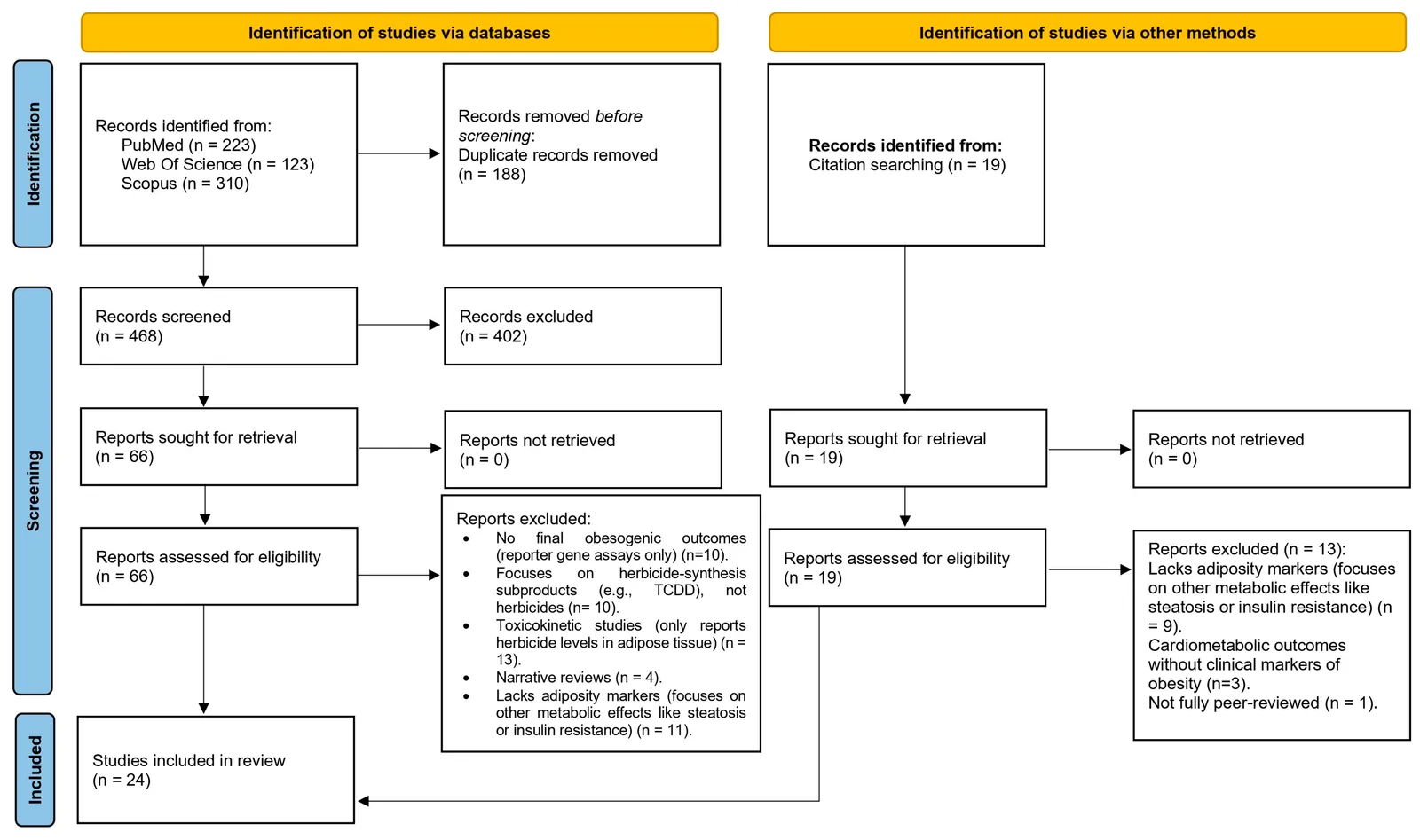
PRISMA-ScR flow diagram detailing the literature search and study selection process.

A total of six hundred and fifty-six records were recovered. After duplicate elimination (*n* = 188), a total of four hundred and sixty-eight records were analyzed. After title screening, four hundred and two were excluded based on an irrelevant title. Next, sixty-six articles were recovered, and nineteen records were identified through reference list analysis. After full-text screening, records were eliminated for five main reasons: (1) obesogenic outcomes were not assessed, (2) the studies focused on subproducts or contaminants linked to herbicide production, (3) the studies reported the herbicide concentrations in adipose tissue, (4) the study was not an original report, and (5) the study included other metabolic outcomes unrelated to obesity. Ultimately, twenty-four records satisfy all eligibility criteria for inclusion (Figure 1).

Table 1 summarizes the epidemiological studies that examined the association between human herbicide exposure and clinical markers of obesity (*n* = 5). All studies were performed in the USA. Eighty percent of the included studies were conducted in the last five years (*n* = 3 in 2025, *n* = 1 in 2023, and *n* = 1 in 2015). One study includes only men, and the remaining studies include both women and men. The prospective cohort and population-based cross-sectional designs were the most frequently studied, with forty percent for each one (*n* = 2). Only one study used an ecological study design. In sixty percent (*n* = 3) of the reviewed studies, herbicides or related metabolites were biomonitored in urine using standardized, globally accepted analytical methods. Forty percent (*n* = 2) of studies estimated the herbicide exposure using structured questionnaires or estimated the county-level herbicide exposure. The assessment of waist circumference or body mass index was used as an obesity-related clinical marker in 80% of included studies (*n* = 4); the rest used the risk of diagnosis of obesity at the country level. The use of methods including computed tomography (the gold standard), magnetic resonance imaging, or indirect methods such as electrical bioimpedance or skinfold anthropometry was not reported. In all studies, multivariate statistical analysis to control for relevant confounders was reported.

Epidemiological evidence has primarily focused on glyphosate, 2,4-D, and atrazine, although the results show little consistency. Some studies using indirect exposure assessments have reported a positive association between atrazine and 2,4-D exposure and obesity. Conversely, urinary levels of atrazine and 2,4-D are inversely related to BMI. Regarding glyphosate, only one ecological study reported a positive association with obesity. Similarly, *in vivo* evidence has mainly concentrated on the obesogenic potential of glyphosate, atrazine, and 2,4-D. Results show greater consistency between glyphosate exposure and elevated obesity-related markers. For atrazine, a transgenerational study reported an anti-obesogenic effect, in contrast to subchronic exposure studies that identified increases in obesity markers. In *in vitro* models, evaluations have predominantly focused on glyphosate: 57.1% of the studies reported inhibition of adipogenesis, while the remainder indicated pro-adipogenic effects. Evidence for other herbicides in *in vitro* models remains scarce, limited to isolated reports.

Table 2 summarizes studies conducted in animal models (*n* = 12). Five included studies (41.6%) were conducted in rats (Sprague-Dawley > Wistar). Four studies (33.4%) were conducted using C57BL/6 mice. Two studies (16.6%) were conducted in Broiler chicks (ROSS 308 strain). Only one study (8.4%) was carried out using zebrafish. Multigenerational (F0-F1) and transgenerational (F0-F3) study designs were reported in four studies (16.6% each).

Commercial formulations were tested in fifty percent of studies, and the rest tested pesticides in analytical or technical grades. Almost all studies reported administering herbicides through oral gavage. Four studies (33.3%) used a two-hit hypothesis (exposure before puberty, exposure to herbicides, and subsequent hypercaloric diets), and exposure to a high-fat diet was the co-treatment most frequently tested after exposure to herbicides. Relative fat mass weight, computed tomographic analysis, and histomorphometric analysis were assessed as obesity biomarkers. Five studies measured the relative expression of key genes involved in adipogenesis in adipose tissue. In seven studies (58.3%), glyphosate was the herbicide tested; in three studies, atrazine was evaluated (25%); and acetochlor and exposure to a mixture of 2,4-D and glyphosate were assessed in one study for each condition. One study (8.4%) reported an intermittent dosing regimen, consisting of herbicide administration for five consecutive days followed by a two-day treatment-free period.

In contrast, the remaining studies reported continuous daily herbicide administration. All included studies employed dose levels derived from LD_50_ estimates. Only one study incorporated a dose equivalent to the acceptable daily intake (ADI); however, none of the studies used doses that reflect environmentally relevant exposure levels.

The evidence from *in vitro* models is presented in Table 3. The murine fibroblast cell line 3T3-L1 was the most widely used cell line for assessing the obesogenic effects of herbicide exposure; primary murine fibroblasts and mesenchymal stromal cells from human and porcine models were also employed. The accumulation of intracellular lipids was used as a biochemical biomarker of adipogenesis; intracellular lipids were assessed using Oil Red O (*n* = 5; 62.5%) or Nile Red (*n* = 3; 37.5%). Glyphosate was the most frequently tested herbicide, and treatment timing was highly inconsistent; dose–response assessment was limited in a few studies. Almost all studies reported only a single dose for the tested herbicide. None of the included studies investigated the effects of herbicide mixtures on obesogenic biomarkers. Four studies (50%) used commercial formulations of herbicides, three studies (37.5%) tested analytical- or technical-grade pesticides, and only one study (12.5%) analyzed the effects of herbicides at technical and commercial grades.

**Figure 2:**
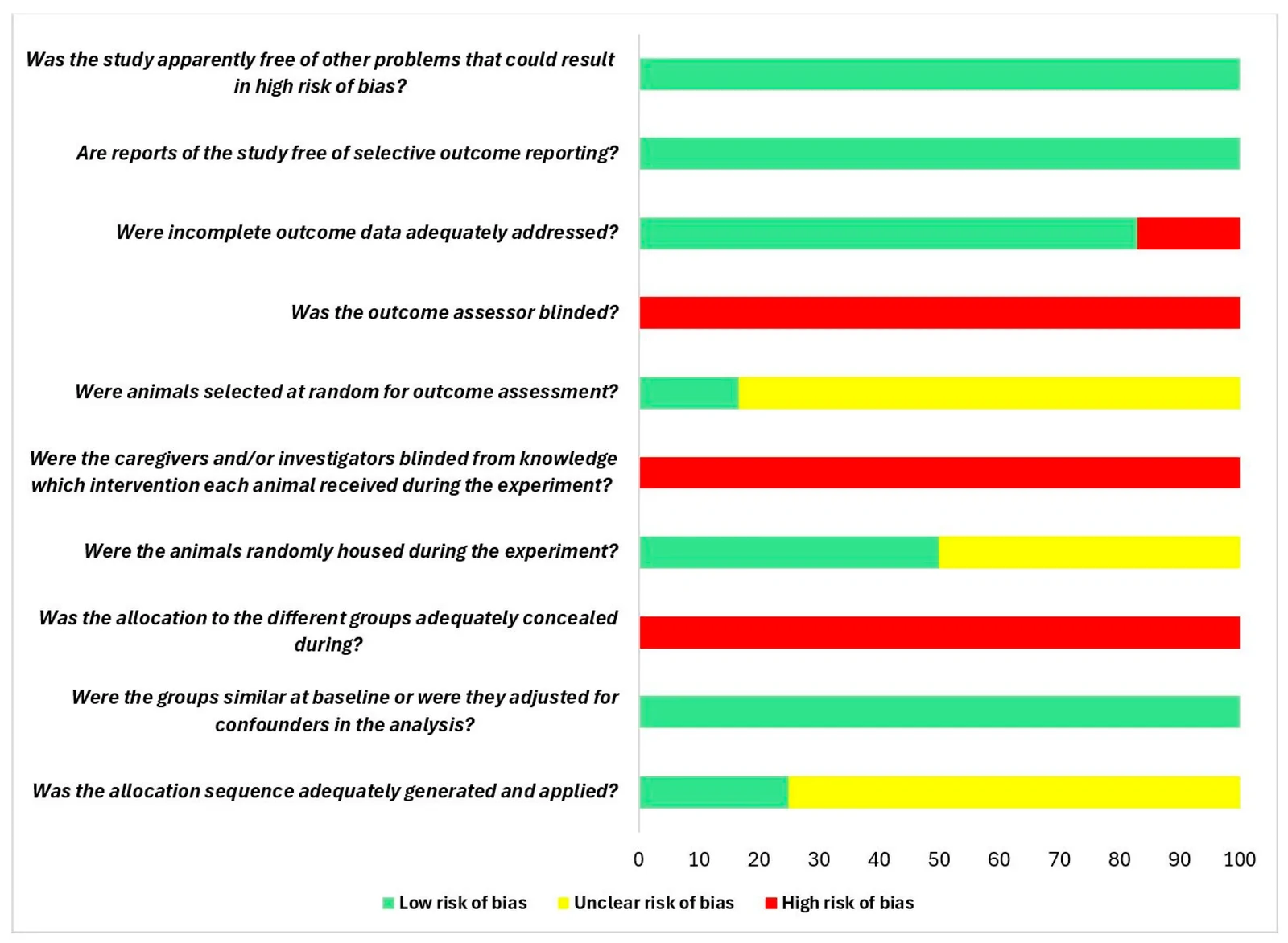
Summary of risk of bias assessment for the included *in vivo* studies using the SYRCLE tool.

Risks of bias from animal studies are summarized in Figure 2. All included studies show a high risk of selection bias (details of intervention allocations were not described). Regarding performance and detection bias, none of the included studies described strategies to blind caregivers to the treatment or control group, and biochemical outcome assessment may have been unblinded (Figure 2).

Table 4 summarizes the risk of bias in epidemiological studies; four included studies (80%) were classified as having a low risk of bias (for each T1 or T2 level). Only one study (20%) was classified as T3 (high risk of bias). Confounding bias was assessed in four studies (80%). The main strength identified in this domain was the use of robust study designs that considered the potential effects of multiple confounders and effect-modifying variables. In addition, all included studies performed statistical analyses adjusted for relevant confounders and effect modifiers. Exposure characterization was judged to have a low risk of bias in two studies (40%). This characterization was based on exposure biomarkers, including measurements of pesticide concentrations or their metabolites. One study characterized exposure by measuring the herbicide or its metabolites in a subset of the included population and estimated exposure based on herbicide-use records (20%). Another study (20%) identified herbicide exposure using validated questionnaires—the study with the highest risk of bias estimated exposure using herbicide sales records and population-based estimates. Finally, obesity-related outcomes were assessed using clinical parameters associated with obesity in all included studies.

## 4. Discussion

The effects of pesticides on the development of several chronic diseases have been studied using epidemiological, animal, and *in vitro* approaches. However, insecticides have been the most investigated. Nevertheless, the effects of herbicides on the development of chronic diseases remain insufficiently studied. This scoping review summarizes the available evidence on the association between herbicide exposure and obesity development.

Preclinical-to-clinical concordance is considered a key topic in toxicology risk assessment. Several issues could be analyzed to assess concordance between preclinical (*in vitro* or *in vivo* studies) and clinical evidence (epidemiological evidence). Three key topics included: (1) the biological relevance associated with the dosage/concentration employed in preclinical studies, (2) the biomarkers measured for assessment of the adverse outcome associated with toxicant exposure, and (3) the representativeness of the preclinical model [37].

The selection of concentrations to be tested in cell culture models to assess the adverse effects of a pesticide compound with regulatory approval for use could be restricted to biologically relevant doses. For a licensed herbicide, this selection may be guided by previously established toxicological reference values, including the acceptable daily intake (ADI), tolerable daily intake (TDI), reference dose (RfD), or acute reference concentration (ARfC). Extrapolation from these values to *in vitro* concentrations is commonly performed using a one-compartment toxicokinetic model, generally assuming a 70-kg adult with approximately 40 L of total body fluid. This approach provides a rationale for defining an experimental concentration range that more closely reflects plausible internal exposure levels, rather than relying exclusively on arbitrary or highly cytotoxic concentrations [38,39]. However, the included studies in this review lack a rationale for selecting the tested concentrations, and selection was limited to non-cytotoxic concentrations obtained from cell viability assays. Unfortunately, this simplified approach fails to account for critical toxicokinetic parameters, including absorption, bioavailability, clearance, and the herbicide’s specific histological tropism. This highlights the limitations of direct dose translation and underscores the need to develop more sophisticated approaches. Future research should prioritize organotropic *in vitro* models and place greater emphasis on physiologically based pharmacokinetic (PBPK) modeling to provide a more accurate translational framework.

For animal studies, the tested doses were lower than one-fifth of the previously reported LD_50_. However, this approach could reduce the extrapolation to human evidence and decrease the similarity of environmentally relevant exposure scenarios. Therefore, future studies should estimate dose–response effects using exposure levels derived from toxicological reference values, such as the ADI, TDI, or RfD. This approach would improve the translational relevance of animal models by more closely reflecting plausible human exposure scenarios [40].

In this regard, the national biomonitoring survey-based studies identified in this review reported concentrations of herbicide or its metabolites in biological matrices. The reported data could be used to estimate relevant experimental doses for animal models through reverse dosimetry. Specifically, herbicide concentrations in blood or metabolite concentrations in urine could be incorporated into physiologically based pharmacokinetic (PBPK) models to estimate the daily external dose to which humans were probably exposed. These estimated doses could then be used as reference points for designing exposure regimens in animal studies [41,42]. Overall, the limited integration of human biomonitoring data, reverse dosimetry approaches, and experimental dose/concentration selection represents one of the main gaps identified in the *in vivo* and *in vitro* studies included in this review. A relevant methodological issue identified in both *in vitro* and *in vivo* studies was the use of either analytical- or technical-grade herbicides, or herbicide-based commercial formulations. Analytical- and technical-grade herbicides allow assessment of the effects associated with the active ingredient.

In contrast, commercial formulations are complex mixtures (which show greater variability across manufacturers) that include inert ingredients that can modify the toxicological profile of the active compound. In realistic exposure scenarios, humans are exposed to commercial herbicide formulations [43,44]. Therefore, assessing commercial formulations may provide a closer approximation to realistic exposure scenarios and improve the environmental and translational relevance of preclinical findings. Furthermore, few studies have analyzed the effects of exposure to a mixture of herbicides; however, several studies show that pesticide use patterns include mixtures of several herbicides, resulting in toxicological interactions (synergists, antagonists, etc.) [44]. Future research could consider exposure to a mixture of herbicides.

The risk-of-bias assessment of the animal studies revealed that none of the included studies implemented caregiver blinding during the experimental procedures. Consequently, caregivers were aware of the allocation of animals to the intervention groups. In addition, outcome assessment was not blinded in any of the studies, which may have increased the risk of performance and detection bias. In this regard, it has been reported that non-blinded caregivers may unintentionally treat animals in the intervention groups differently from those in the control groups. Furthermore, the lack of blinding during outcome assessment, including biomarker evaluation, may lead to an overestimation of treatment effects and an increased likelihood of false-positive findings [45]. Therefore, blinding of both the intervention procedures and the outcome assessments is essential to reduce bias and ensure the validity of the experimental findings.

Another key issue to consider is that all epidemiological studies examining the relationship between herbicide exposure and obesity-related clinical biomarkers were conducted in the USA. Although two studies were based on national population surveys that included multiracial participants, and one study included a cohort predominantly composed of individuals with Mexican ancestry, the lack of available studies in populations of Asian or African ancestry, as well as the absence of data from developing countries, limits external validity and highlights the need to conduct epidemiological studies in other countries. In addition, animal studies have included transgenerational, multigenerational, and two-hit exposure models. However, epidemiological studies on herbicide exposure and obesogenic effects have only reported cross-sectional designs. For other pollutants, evidence derived from animal and epidemiological studies has addressed transgenerational effects and susceptibility windows of exposure [46,47,48]. This indicates that research on herbicide-induced obesity in animal models remains poorly connected with epidemiological studies. There is a cohort that includes children and parents, such as the Center for the Health Assessment of Mothers and Children of Salinas (CHAMACOS) study. In this line, epigenetic obesity markers could be assessed in parents (F0) and their sons (F1), and analysis of epigenetic obesity markers in F1 sperm could reveal transgenerational effects [47].

Translating evidence from *in vitro* and *in vivo* models to human findings requires that the endpoints assessed in cells and animal models be biologically and clinically relevant. In this regard, intracellular lipid accumulation (considered a reference standard for evaluating adipogenesis *in vitro)* is not a representative model of the pathophysiology of human obesity. In addition, the expression of master regulators of adipogenesis, such as PPARγ or SREBP, in *in vitro* systems and animal models may not correlate with their behavior in humans [37]. Furthermore, as previously mentioned, some epidemiological studies have included only men, a limitation also observed in experimental models that exclusively employ males or females. This hinders translational toxicology, as biological sex modifies the toxicokinetics and toxicodynamics of environmental xenobiotics, including herbicides. Differences in body composition, such as fat distribution and total body water proportion, can alter toxicokinetics. Likewise, the expression and activity of xenobiotic-metabolizing enzymes exhibit marked sexual dimorphism, while hormonal fluctuations can alter toxicodynamic responses. Consequently, *in vivo* and epidemiological study designs mustn’t be restricted to a single biological sex [49].

Finally, epidemiological studies primarily use body mass index as a clinical indicator of obesity; however, several studies have shown that, under conditions of increased muscle mass, BMI is susceptible to bias. Other clinical markers include abdominal obesity or body composition data obtained through bioelectrical impedance measurements, which show a high correlation with methods considered the gold standard, such as computed tomography, for estimating fat mass [50]. Nevertheless, the aforementioned obesity-related markers in individuals exposed to herbicides have not been reported.

The findings of this scoping review indicate that the *in vitro* studies are highly heterogeneous in experimental design, exposure conditions, and dose ranges, thereby precluding a meaningful meta-analysis. Similarly, most of the epidemiological evidence is derived from analyses of the NHANES database in the United States. Consequently, the assumption of data independence may not be fully met across studies, potentially limiting the representativeness and validity of a pooled meta-analysis.

In contrast, animal studies exhibit greater methodological consistency, including comparable exposure doses and similar endpoints for obesity assessment. Therefore, conducting a meta-analysis of animal studies appears feasible and could provide a more robust quantitative estimate of the overall effect, thereby strengthening the external validity of the available evidence. Furthermore, meta-regression analyses could be performed to evaluate potential dose–response relationships and to explore the influence of exposure levels on obesity-related outcomes.

Future research should address several methodological gaps identified across the current scoping review. For *in vitro* models, greater emphasis should be placed on selecting exposure levels. The concentrations for testing could be selected considering realistic human and environmental exposure scenarios. Future studies should consider doses informed by established toxicological reference values, such as the ADI and ARfD. Moreover, real environmental or occupational exposure occurs through herbicide mixtures; therefore, the analysis of herbicide mixtures could be performed. For *in vivo* studies, rigorous implementation of blinding procedures is essential. Both the allocation of animals to intervention groups and the assessment of end-outcome results should be conducted under blinded conditions to minimize the risk of effect overestimation and reduce the likelihood of false-positive findings. In addition, the growing epidemiological evidence describing urinary concentrations of herbicides and their metabolites in environmentally exposed populations provides an opportunity to improve the translational relevance of animal experiments. Specifically, reverse dosimetry approaches could be employed to estimate biologically relevant internal doses, which may then be used to define exposure levels for experimental animal studies. Finally, future epidemiological research should move beyond the widespread reliance on BMI as the primary measure of obesity. Incorporating more sensitive and specific indicators of adiposity, body composition, and metabolic dysfunction would likely improve the identification of obesity-related effects associated with herbicide exposure. While we identified cohort, ecological, and national survey studies, most relied heavily on cross-sectional designs. We recommend shifting toward multigenerational and transgenerational investigations to understand the long-term, heritable consequences of herbicide exposure on obesity. Additionally, ecological and survey-based studies should account for temporal fluctuations in herbicide application, sales, and exposure. Future approaches must also evaluate the synergistic, additive, or antagonistic interactions between herbicide exposure and intrinsic or lifestyle factors, including physical activity, urbanization, and dietary changes, which would provide a more comprehensive understanding of the multifactorial nature of herbicide-induced obesogenesis.

The findings of this scoping review should be interpreted in light of several limitations. First, most *in vitro* studies report non-standardized methodological data, hindering the assessment of risk of bias. Second, there is evidence of overlap in epidemiological data, particularly among studies based on national surveys. Third, this review was restricted to studies published in English; therefore, gray literature or research in other languages may have been overlooked. Finally, the high heterogeneity of study designs across *in vitro*, *in vivo*, and epidemiological research warrants a cautious interpretation of the obtained results.

## Figures and Tables

**Table 1:** Methodological characteristics of the included epidemiological studies evaluating herbicide exposure and obesity-related clinical markers.

Study	Study Design	Country	Sample Size	Sex	Age	Target Population	Time of Exposure	Exposure Assessment					Outcome Assessment		Main Findings
								Identified Herbicides	Biomarker or Pesticide Assessed	Method	Matrix	Other	Obesity-Related Clinical Markers	Measurement Method	
Eskenazi et al. [13]	PC	USA	480	F/M	18 years	CHAMACOS cohort (mother-child dyads)	18 yrs	GLY	U-GLY/SG U-AMPA/SG	UPLC-MSMS	Urine	Agricultural GLY applied within 1-km residential radius	Large waist circumference ≥40 inches for males or ≥35 inches for females	Direct measurement via physical examination	In 18-year-olds (*n* = 72), large waist circumference was associated with total GLY residues (RR = 1.33; 95%CI: 1.02–1.73) only in those with 2-fold increases in urinary GLY, AMPA, and total residue concentrations
He et al. [14]	NPB-CS	USA	12,132	F/M	20 to ≥60 years	Individuals without CVD-related mortality	NA	2,4-D, ATZ, GLY	U-2,4-D and met/CrU-ATZmet/CrU-GLY/Cr	IC-ID-MS/MS	Urine	QNR	BMI score	Direct measurement via physical examination with categorical scoring	An inverse relationship was observed between the BMI score and both U-2,4-D (β = −1.4; *p* < 0.01) and U-ATZmet (β = −1.18; *p* < 0.01).
Feng et al. [15]	NPB-CS	USA	2094	F/M	48.61 ± 18.28 years	General population	NA	GLY	U-GLY/Cr	IC-ID-MS/MS	Urine	QNR	BMI, WC	Direct measurement via physical examination	Quartile-stratified U-GLY levels were not associated with changes in WC or BMI.
LaVerda et al. [16]	PC	USA	8365	M	20 to ≥60 years	Licensed private pesticide applicators	Cumulative exposure days (mostly ≥20 years)	EPTC, 2,4-D, 2,4,5-T, 2,4,5-TP, ATZ, DIC, CYA, CHL, MET, ALA, MBZ, PQ, PEND, IMZ, GLY, BUT, TRI	NA	NA	NA	Self-report-QNR	BMI	Direct measurement; categorized as normal (18.5–<25), overweight (25–<30), and obese (≥30).	A dose–response trend was observed for overweight/obesity ORs with triazine and atrazine herbicides, though only the highest exposure group was statistically significant.
Otaru et al. [17]	ECO	USA	18,382	F/M	20 to ≥60 years	Country level	NA	2,4-D, ATZ, DIC, TRI, ACE, CTL, DIM-P, GLUF, PEN, PQ, MET, S-MET, GLY	NA	NA	NA	Exposure estimates	County-level adult obesity prevalence	County-level adult obesity (%) (Data source: CDC BRFSS; estimated via multilevel modeling)	County-level obesity was associated with per-hectare application rates of 2,4-D, ATZ, ACE, GLUF, PEN, MET, and S-MET and GLY

Study design: ECO, ecological study; NPB-CS, national population-based cross-sectional study; PC, prospective cohort. Sex: F, female; M, male. Herbicides: ACE, acetochlor; ALA, alachlor; ATR, atrazine; BUT, butylate; CHL, chlorimuron; CYA, cyanazine; DIC, dicamba; DIM-P, dimethenamid-P; EPTC, S-ethyl dipropylthiocarbamate; GLUF, glufosinate; GLY, glyphosate; IMZ, imazethapyr; MBZ, metribuzin; MET, metolachlor; PEN, pendimethalin; PQ, paraquat; TRI, trifluralin. Exposure biomarkers: AMPA, aminomethylphosphonic acid; U-AMPA/SG, urinary aminomethylphosphonic acid standardized by specific gravity; U-ATZmet/Cr, creatinine-adjusted urinary atrazine metabolites; U-GLY/Cr, creatinine-adjusted urinary glyphosate; U-GLY/SG, urinary glyphosate standardized by specific gravity; U-2,4-D/Cr, creatinine-adjusted urinary 2,4-dichlorophenoxyacetic acid. Analytical methods: IC-ID-MS/MS, ion chromatography isotope dilution tandem mass spectrometry; UPLC-MS/MS, ultra-performance liquid chromatography–tandem mass spectrometry. Cohorts and data sources: BRFSS, Behavioral Risk Factor Surveillance System; CDC, Centers for Disease Control and Prevention; CHAMACOS, Center for the Health Assessment of Mothers and Children of Salinas. Other abbreviations: QNR, questionnaire; WC, waist circumference.

**Table 2:** Experimental design and obesogenic endpoints of the included *in vivo* studies assessing herbicide exposure in animal models.

Study	Animal Model	Sample Size	Sex	Age	Study Design	*In Vivo* Exposure Protocol							Obesity Assessment	Main Findings
						Herbicide		Dosage	Vehicle	ROA	Exposure Frequency/Duration	Concurrent Treatment	Outcome	
						Active Ingredient	Purity							
de Paula Xavier et al. [18]	C57Bl/6 mice	*n* = 6–11 per group	F	100 days	Sub-chronic	GLY	Commercial formulation	0.5 and 50 mg/kg bww	Distilled water	Oral gavage	Daily for 60 days	Ovariectomy and ovary experorizaion	Gravimetric and histomorphometric analysis of BAT (interscapular) and WAT (inguinal, periuterine). Relative mRNA expression of *Ppar-γ* and *Prdm16* in interscapular BAT.	GLY (50 mg/kg/day) induced BAT lipid droplet hypertrophy (indicating a low-thermogenic phenotype) and increased *Ppar-γ* expression in OVX females, highlighting a dependence on ovarian hormone status.
Estienne et al. [19]	F0: ROSS 308 breeder hens; F1: Broiler chicks	F1 *n* = 399 (213 exp./186 unexp.)	F/M	Post-hatch days (PHD) 5 and 10	*In vivo* multigenerational study (F0–F1)	GLY	Commercial formulation	47 mg/kg bww	Food	Oral	Daily for 6 weeks	Maternal exposure (6-week period prior to laying)	Relative weight of subcutaneous and abdominal adipose tissue (F1). Relative expression of Chemerin/CMKLR1 from adipose tissue (F1).	Progeny of GLY-exposed hens exhibited increased relative abdominal fat mass compared to controls.
Fréville et al. [20]	Broiler chicks (ROSS 308 strain)	*n* = 75/per group	F	32 weeks old	Sub-chronic	GLY	Commercial formulation	47 mg/kg bww	Food	Oral	Daily for 6 weeks	NA	Relative weight of abdominal adipose tissue assessed at termination and following a 4-week washout period.	No significant differences in relative abdominal adipose tissue weight were observed among experimental groups.
Lim et al. [21]	Sprague-Dawley rats	*n* = 48	M	8 weeks old	Chronic	ATR	NA	30 and 300 μg/kg bww	Drinking water	Oral	Daily for 5 moths	Normal or high-fat diet	Non-contrast CT quantification of visceral adipose tissue (VAT) area at L3 and L1–L5 levels.	Obesity markers were higher in the exposed group compared to controls, though no dose–response relationship was observed.
Lima et al. [22]	Wistar rats	*n* = 8–12/per group	F/M	F0: 40 days old F1: 70 days old	*In vivo* multigenerational study (F0–F1)	GLY	Commercial formulation	0.5 and 50 mg/kg bww	Drinking water	Oral gavage	Daily for 21 days	Maternal Gly-exposure (pregnancy −21 days; F0). F1: DEX (1 mg/kg bw) or saline vehicle (1 mL/kg bw); i.p.; once daily for 6 d.	Adipocyte size and histomorphometric analysis from perigonadal adipose tissue	GLY exposure had no significant effect on the analyzed markers.
Kubsad et al. [23]	Hsd:Sprague Dawley^®^	*n* = 100	F/M	F0: 70–100 days old	*In vivo* transgenerational study (F0–F3)	GLY	Technical grade	F0: 25 mg/kg bww	PBS	intraperitoneal	Daily for 7 days (Exposure from GD 8 to 14)	NA	Hypertrophic adipocyte area; phenotype stratification (Obese/lean cutoff at +1.5 SD from control mean).	Increased obesity incidence was observed in both male and female F2 and F3 offspring of GLY-exposed F0 rats, suggesting transgenerational effects.
McBirney et al. [24]	Hsd:Sprague Dawley^®^	*n* = 100	F/M	F0: 70–100 days old	*In vivo* transgenerational study (F0–F3)	ATR	Technical grade	F0: 25 mg/kg bww	DMSO	intraperitoneal	Daily for 7 days (Exposure from GD 8 to 14)	NA	Hypertrophic adipocyte area; phenotype stratification (Obese/lean cutoff at +1.5 SD from control mean); and DNA-normalized fat pad weight.	An increased frequency of a lean phenotype was observed in both male and female F3 offspring of atrazine-treated groups compared to controls.
Mesmar et al. [25]	Wild-type NHGRI-1 fish (Zebrafish)	*n* = 20–30 per group	NA	48 and 72 hpf	Acute	ACE	Analytical grade	0.78 and 7.8 µM	DMSO	Waterborne exposure	24 h.	NA	Transcriptomic profiling via RNA-seq (assembly, DEG identification, and adipogenic pathway enrichment).	Transcription factor enrichment analysis predicted the upregulation of adipogenic pathways, including those regulated by CEBPA and CREB3L3
Rakshitha et al. [26]	Wistar-rat	*n* = 6 per group	M	NA	Sub-chronic	GLY	Technical grade	50, 100 and 250 mg/kg bww	Drinking water	Oral gavage	Daily for 16 weeks	NA	Relative mRNA expression of Srebp-1c and Ppar-γ in adipose tissue.	An increase in Srebp-1c and a reduction in Ppar-γ were observed, with changes following a dose–response trend.
Romualdo et al. [27]	C57BI/6 J mice	*n* = 10/group	M	5–6 weeks old	Chronic	GLY; 2,4.D and the mixture	Technical grade	Gly (0.05 and 5 mg/kg bw); 2,4-D (0.02 and 2 mg/kg bw). Mixtures: Low Gly-2,4-D (0.05:0.02 mg/kg bw) Hight Gly-2,4-D (5:2 mg/kg bw)	Ultrapure distilled water	Intragastrical administration	5 days per week	Hypercaloric high-sugar fat diet and a high sugar drinking solution.	Absolute and relative weights of visceral (mesenteric, retroperitoneal) and subcutaneous adipose tissue depots.	Herbicide-exposed groups exhibited higher absolute and relative weights of visceral and subcutaneous adipose tissue.
Rosolen et al. [28]	C57Bl/6 mice	*n* = 20F or 25M/per group	F/M	21 days old	Subacute	GLY	Commercial grade	50 mg/kg bww	Wate	Oral gavage	Daily for 4 weeks	After the glyphosate exposure a high fat diet was administrated	Histomorphometry of iBAT, perigonadal, and retroperitoneal WAT; alongside chemical body composition via carcass ether extraction (relative fat and lean mass %).	Female mice exhibited increased perigonadal and subcutaneous fat alongside reduced lean mass. While both sexes in the GLY-HFD group developed hypertrophic white adipocytes, pubertal GLY exposure exacerbated HFD-induced fat accumulation in brown adipocytes exclusively in females.
Teleken et al. [29]	C57Bl/6 mice	*n* = 6/group	F/M	21 days old	Subacute	ATR	Commercial grade	5 mg/kg bww	Distilled water	Oral gavage	Daily for 30 days	Peripubertal atrazine exposure followed by a 64-day high-fat diet (HFD) challenge.	Histomorphometry of iBAT, perigonadal, and retroperitoneal WAT; alongside chemical body composition via carcass ether extraction (relative fat and lean mass %). Relative mRNA expression of Srebp-1c and Ppar-γ in adipose tissue.	Despite no changes in body weight or subcutaneous/total WAT mass, ATZ-exposed males showed adipocyte hypertrophy and upregulated Srebp-2, and Ppar-γ in perigonadal WAT

Abbreviations: F, female; M, male. Animal models: Hsd, Harlan Sprague–Dawley; SD, standard deviation. Study design: F0, parental generation; F1, first filial generation; F3, third filial generation. Herbicides: ATR, atrazine; GLY, glyphosate. Exposure: bww, body weight; DEX, dexamethasone; DMSO, dimethyl sulfoxide; GD, gestational day; i.p., intraperitoneal; NA, not available; PBS, phosphate-buffered saline; ROA, route of administration; hpf, hours post-fertilization; RNA-seq, RNA sequencing; DEG, differentially expressed gene; WAT, white adipose tissue; iBAT, interscapular brown adipose tissue; HFD, high-fat diet; DMSO, dimethyl sulfoxide. Herbicides: ATR, at-razine; GLY, glyphosate.

**Table 3:** Summary of *in vitro* evidence evaluating the effects of herbicide exposure on adipogenesis.

Study	Cell Line	*In Vitro* Exposure Protocol					Adipogenic Assessment	Main Findings
		Herbicide					Outcome	
		Active Ingredient	Concentration	Vehicle	Time of Exposure	Purity		
Biserni et al. [30]	Murine fibroblast 3T3-L1	Quizalofop-p-ethyl	0.1–100 µM	DMSO	6 days	Analytical grade	Intracellular lipid staining (Nile red), activation of PPARγ-responsive promoter, and RNA-seq analysis of KEGG pathways	Quizalofop-p-ethyl induced dose-dependent intracellular lipid accumulation. GLY, 2,4-D, isoxaflutole, dicamba, and commercial quizalofop showed similar, but non-dose-dependent, effects. Additionally, RNA-seq enrichment analysis indicated the upregulation of adipogenic pathways following 24-h quizalofop-p-ethyl exposure. Furthermore, quizalofop-p-ethyl was identified as an inducer of PPARγ expression.
		GLY	0.1–100 µM					
		2,4-D	0.1–100 µM					
		Isoxaflutole	0.1–100 µM					
		Mesotrione	0.1–100 µM					
		DIC	0.1–1000 µM					
		Quizalofop	0.1–1000 µM					
		Propaquizafop	0.1–1000 µM					
		Quizalofop-formulation	0.1–1000 µM					
de Melo et al. [31]	Primary culture of human adipose-derived mesenchymal stem cells (hASCs),	GLY	36 μg/mL	NA	21 days	Commercial formulation	Intracellular lipid staining (Oil Red O) and PPARG2 expression.	GLY exposure induced an increase in intracellular lipid accumulation compared to the control group; however, no changes were observed in the relative expression of *PPARG2*.
Gigante et al. [32]	Primary culture of porcine adipose stromal cells (ASCs) from swine adipose tissue	GLY	4 μg/mL	NA	12 days	Technical grade	Intracellular lipid staining (Oil Red O) and PPARG2 expression.	Glyphosate inhibited ASC adipogenic differentiation, with no effect on relative PPARγ expression.
Janesick et al. [33]	Murine fibroblast 3T3-L1	ACE ALA Asulam Flumetsulam Maleic hydrazide	0.02, 0.2, 2, and 20 μM	DMSO	5 days	Technical grade	Intracellular lipid staining (Nile Red) and expression of Zfp423	ACE and ALA did not induce adipogenesis but acted as PPARγ antagonists; the remaining tested herbicides showed neither adipogenic effects nor effects on PPARγ.
Martini et al. [34]	Murine fibroblast 3T3-L1	GLY	≈178 mg/mL	Medium supplemented	8 days	Commercial formulation	Intracellular lipid staining (Oil Red O)	GLY exposure inhibited adipogenesis when cells were co-exposed to GLY and the adipogenic induction medium. However, if cells were first exposed to glyphosate followed by the induction medium, adipogenic differentiation was restored.”
Martini et al. [35]	Murine fibroblast 3T3-L1	GLY	≈36, 89, and 178 mg/L	Medium supplemented	8 days	Technical grade and commercial formulation	Intracellular lipid staining (Oil Red O)	Glyphosate-based herbicides containing both polyethoxylated and non-polyethoxylated adjuvants inhibited adipocyte differentiation
Martini et al. [36]	Murine fibroblast 3T3-L1 and primary culture of mouse embryonic fibroblasts	GLY	≈178 and 89 mg/L	Medium supplemented	8 days	Commercial formulation	Intracellular lipid staining (Oil Red O) and expression of *PPARγ* and *C/EBPβ*	A commercial glyphosate-based herbicide inhibited proliferation and adipogenesis in 3T3-L1 and MEFs due to the downregulation of the master adipogenic gene Pparγ, but not C/ebpβ.
Mesmar et al. [25]	Murine fibroblast 3T3-L1	ACE	10 µM	DMSO	8 days	Technical grade	Intracellular lipid staining (Oil Red O and Nile Red) and expression of *PPARγ*	Acetochlor increased intracellular lipid content without a clear dose-response relationship and upregulated PPARγ expression

Abbreviations: Cell lines: 3T3-L1, murine preadipocyte cell line; ASCs, adipose stromal cells; hASCs, human adipose-derived mesenchymal stem cells. Herbicides: ACE, acetochlor; ALA, alachlor; DIC, dicamba; GLY, glyphosate. Vehicles: DMSO, dimethyl sulfoxide; NA, not available. Adipogenic assessment: KEGG, Kyoto Encyclopedia of Genes and Genomes; RNA-seq, RNA sequencing; PPARγ, peroxisome proliferator-activated receptor gamma; PPARG2, peroxisome proliferator-activated receptor gamma 2; C/EBPβ, CCAAT/enhancer-binding protein beta; Zfp423, zinc finger protein 423.

**Table 4:** Risk of bias assessment for the included epidemiological studies using the OHAT tool.

**Study**	**Selection Bias**	**Confounding Bias**		**Detection Bias**				SUMMARY TIERED CLASSIFICATION
	Were the Comparison Groups Appropriate?	**Did the Study Design or Analysis Account for Important Confounding and Modifying Variables?**	Did Researchers Adjust or Control for Other Exposures That Are Anticipated to Bias Results?	Were the Outcome Assessors Blinded To Study Group Or Exposure level?	Were Confounding Variables Assessed Consistently across groups using valid and reliable measures?	**Can We Be Confident In the Exposure Characterization?**	**Can We Be Confident in the Outcome Assessment?**	
Eskenazi et al. [13]	**++**	**++**	**++**	**-**	**++**	**+**	**++**	***T2***
He et al. [14]	**++**	**++**	**++**	**+**	**++**	**++**	**++**	***T1***
Feng et al. [15]	**++**	**++**	**++**	**+**	**++**	**++**	**++**	***T1***
LaVerda et al. [16]	**+**	**++**	**++**	**+**	**++**	**-**	**++**	***T2***
Otaru et al. [17]	**+**	**+**	**++**	**+**	**++**	**--**	**++**	***T3***

**Abbreviations:** OHAT, Office of Health Assessment and Translation. Risk of bias ratings: ++: Definitely low risk of bias; +: Probably low risk of bias; --: Definitely high risk of bias; -: Probably high risk of bias.

## Data Availability

No new data were created or analyzed in this study. Data sharing is not applicable to this article.

## Supplementary Materials

The following supporting information can be downloaded at: https://www.mdpi.com/article/10.3390/jox16050173/s1.
