# Supplementary material for: Translational Gaps in Assessing the Obesogenic Effects of Herbicides: A Scoping Review
Source: J Xenobiot. 2026 Sep 13;16(5):173. doi: 10.3390/jox16050173 (PMC13608544; doi:10.3390/jox16050173)
Supplement: Supplementary file 1 [file jox-16-00173-s001.zip › jox-4516033-supplementary material two.pdf]

Translational Gaps in Assessing the Obesogenic Effects of Herbicides: A Scoping Review

Marco Antonio Ramírez-Vargas, Cristian Oswaldo Bravo-Nava, Ma. Elena Moreno-Godínez, Gerardo Huerta-Beristain, Eugenia Flores-Alfaro, Guillermina Vences-Velázquez and José Ángel Cahua-Pablo

1. PubMed

```
( "Herbicides"[MeSH] OR herbicide*[tiab] ) AND ( "Adipogenesis"[MeSH] OR "Adipose
Tissue"[MeSH] OR "Adipocytes"[MeSH] OR "Cell Differentiation"[MeSH] OR
adipogenes*[tiab] OR adipocyt*[tiab] OR "adipose tissue"[tiab] ) )
```

2. Scopus

```
herbicide*

AND

adipogenes* OR adipocyt* OR "adipose tissue" OR "fat tissue" OR "white adipose
tissue" OR "brown adipose tissue" OR "cell differentiation"
```

3. Web of Science (WoS)

```
herbicide* (Topic)

AND

( adipogenes* OR adipocyt* OR "adipose tissue" OR "fat tissue" OR "white adipose
tissue" OR "brown adipose tissue" OR "cell differentiation" ) (Topic)
```
